# Diminished Frontal Theta Activity During Gaming in Young Adults With Internet Gaming Disorder

**DOI:** 10.3389/fnins.2019.01183

**Published:** 2019-11-01

**Authors:** Juri Kim, Jinsick Park, Young Min Park, DongPyo Jang, Kee Namkoong, Young-Chul Jung, In Young Kim

**Affiliations:** ^1^Department of Biomedical Engineering, Hanyang University, Seoul, South Korea; ^2^Institute of Behavioral Science in Medicine, Yonsei University College of Medicine, Seoul, South Korea; ^3^Department of Psychiatry, Yonsei University College of Medicine, Seoul, South Korea

**Keywords:** EEG, left frontal cortex, cognitive control, theta power, internet gaming disorder

## Abstract

Cognitive control is essential for flexible, top-down, goal-directed behavior. Individuals with Internet gaming disorder (IGD) are characterized by impaired prefrontal cortex function and cognitive control. This results in an increase in stimulus-driven habitual behavior, particularly related to pathological gaming. In the present study, we investigated the electroencephalographic (EEG) activity in individuals with IGD. Twenty-four individuals with IGD and 35 healthy control (HC) subjects were recruited. We analyzed their EEG activity while the subjects played their favorite game (30–40 min duration). We compared the band power between the two groups. During gaming, the left frontal theta, alpha, and beta band activities were lower in subjects with IGD than in HCs. Moreover, the left frontal theta power negatively correlated with IGD severity. These results indicate that left frontal theta power could be used as a neurophysiological biomarker for the detection of diminished cognitive control patterns in individuals with IGD.

## Introduction

Internet gaming disorder (IGD) is a specific form of Internet addiction characterized by an individual’s impaired control over Internet gaming (Kuss, 2013). As with other addictions, individuals with IGD exhibit salience, mood modification, tolerance, withdrawal symptoms, conflicts, and relapses (Chou and Ting, 2003). In the recent 11th Revision of the International Classification of Diseases (ICD-11) from the World Health Organization (WHO), a gaming disorder is defined as a pattern of gaming behavior (“digital-gaming” or “video-gaming”) characterized by an impaired control over gaming. Increasing priority is given to gaming over other activities to the extent that gaming takes precedence over other interests and daily activities. The continuation or escalation of gaming persists despite the occurrence of negative consequences (Young, 2018).

Many studies have demonstrated the neurophysiological features of IGD using electroencephalography (EEG). Most previous studies have focused on identifying biomarkers for subjects with IGD in the resting state. A study by Choi et al. (2013) revealed that subjects with IGD had a lower beta band power in all regions and a higher gamma band power in the frontal regions than those of healthy control (HC) subjects. These results were related to impulsivity. In addition, Lee et al. (2014) demonstrated that the absolute beta band power in all brain regions was lower in subjects with IGD who did not have depression, than in those with depression and in HC subjects. The lower beta activity is correlated with the impulsivity and dysfunctional inhibitory control in subjects with IGD without depression. Park et al. reported that the intra-hemispheric coherence values for the theta band between the T4-T6 and P4-O2 electrodes were higher in subjects with attention-deficit/hyperactivity disorder (ADHD) with comorbid IGD than those in subjects with ADHD without comorbidity (Park et al., 2017). They indicated that the repetitive activation of the brain reward system during continuous gaming may have increased the neuronal connectivity within the parieto-occipital and temporal regions in the subjects with ADHD alone, compared to those with comorbid IGD. These studies provided evidence that the excessive use of Internet games results in cognitive impairment and functional changes in the brain of subjects with IGD.

To prevent the cognitive impairment caused by IGD, it is necessary to monitor the cue-induced neurophysiological responses during gaming. It is assumed that gaming may be associated with excessive rewarding behavior that meets the criteria of addiction. Gaming can elicit a strong motivational state that contributes to cravings and repeated excessive or addictive behaviors (Thalemann et al., 2007). For example, in a study investigating pathological gambling, a significant increase in the mean craving for gambling was found in subjects with pathological gambling who were exposed to visual gambling cues during the assessment period (Crockford et al., 2005). In an IGD study on the cue-reactivity paradigm, the differences in the cue-induced event-related potentials between excessive computer-game players and casual players were significant for game related-cues. However, they were not significant for non-game related cues (Thalemann et al., 2007). Yao et al. (2015) reported inhibition deficits during the performance of a gaming-related Go/No-Go task in subjects with IGD compared with HCs. However, the IGD group did not differ from the HC group in non-gaming-related inhibitory control, as assessed by the Stroop task.

Previous studies have demonstrated that the an addiction cue can lead to strong motivation. This has led to studies that have examined the differences before and after an addiction cue. However, the aforementioned studies did not describe the gaming cue-induced neurophysiological response during gaming in individuals with IGD. A recent study investigated the changes in the heart rate variability (HRV) patterns during gaming as a potential biomarker for IGD (Hong et al., 2018; Lee et al., 2018). Compared with HCs, subjects with IGD exhibited an altered HRV response while playing an online game. This indicates that the dynamics between executive control and reward-seeking may be out of balance during gaming in these subjects. This prompted us to initiate a study of the cue-induced neurophysiological responses during gaming. EEG can be used to measure the neurophysiological responses in real time with a higher temporal resolution than that of fMRI. In addition, EEG can be correlated with cognitive functions and can measure the neural activity directly, with a multidimensional signal encompassing time, space, frequency, and power (Cohen, 2011). In this study, we used EEG to analyze the cue-induced neurophysiological responses during gaming.

We hypothesized that the differences in the brain functions between individuals with and those without IGD would become apparent during gaming. Furthermore, if the activity in specific regions in the brain would be significantly different between the groups, this would be a beneficial biomarker that could be used to classify IGD. Thus, in this study, we examined the changes in the power of the theta, alpha, and beta bands between the resting state and the gaming state using EEG. We compared them between individuals with and those without IGD to determine their significance. The purpose of this study was to clarify whether the brain activity changed during gaming in individuals with IGD without other psychological problems (e.g., depression and anxiety). Since previous studies have reported a difference in motivation between the resting state and after a game-related cue in a cue-related task in IGD, we further investigated the difference in the brain activity while gaming compared with that at the resting state between the IGD and HC groups.

## Materials and Methods

### Participants

We recruited 59 right-handed young males. The subjects were examined for their Internet usage patterns and were administered the Young’s Internet Addiction Test (YIAT) (Beard and Wolf, 2001; Young, 2011). Subjects who used the Internet primarily for gaming and whose YIAT scores were more than 50, were classified as the IGD group (*n* = 24, mean age = 23.3 ± 2.3 years). They were confirmed to have IGD by a psychiatrist according to the IGD diagnostic criteria of the DSM Fifth Edition (Petry and O’Brien, 2013). Subjects who scored below 50 on the YIAT were classified as HCs (*n* = 35, mean age = 23.2 ± 2.5 years). All subjects in this study frequently played “League of Legends (LOL),” (Riot Games, Los Angeles, CA, United States, 2009). This is the most popular multiplayer online battle arena game in Korea (Nuyens et al., 2016, Statista, 2018). To control for differences in skill between the IGD group and the HC group, we included only those who were ranked above the silver tier. There were six tiers in this game, bronze, silver, gold, platinum, diamond and challenger.

All subjects completed several self-reporting questionnaires assessing comorbid psychiatric symptoms of IGD (Kim et al., 2016). The following self-reporting questionnaires were used:

1. The Beck Depression Inventory to test for depression (Beck et al., 1961).
2. The Beck Anxiety Inventory to test for anxiety (Beck et al., 1988).
3. The Barratt Impulsiveness Scale, version 11 to evaluate impulsivity (Patton et al., 1995).
4. The Alcohol Use Disorders Identification Test to identify alcohol-related problems (Reinert and Allen, 2002).
5. The Wender Utah Rating Scale to evaluate for symptoms of ADHD (Ward et al., 1993).

### Task and Procedure

**FIGURE 1:**
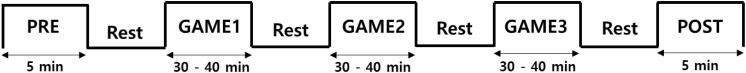
Experimental protocol for recording scalp EEG signals.

Resting-state EEG (PRE) was recorded with the participants in a comfortable sitting position with their eyes open for 5 min (Figure 1). Each subject then played the online game LOL three times, (periods GAME1, GAME2, and GAME3) with 5 min of rest between gaming periods. Following the gaming, resting-state EEG was again recorded for 5 min (POST). The Institutional Review Board approved the protocol for this study (HYI-16-044), and all subjects provided signed informed consent before participating.

### EEG Data Acquisition and Pre-processing

EEG data were acquired using a Waveguard 64 EEG sensor-cap (CA-105, ANT-Neuro, Enscheda, Netherlands). The signals were sampled at a frequency of 1024 Hz and we used the active G1/G2 ground reference (BRAINBOX EEG-1166 system, Braintronics, Almere, Netherlands) for the ground and reference (G1: FPz, G2: AFz). As a reference, a single channel with bipolar electrodes was attached to the mastoids. Possible line noise artifacts were removed from the data with a notch filter at 60 Hz in the central frequency, IIR notch filter in filter type, and 9.9 Hz in bandwidth. The impedance of each electrode was maintained below 10 kiloohms.

Artifact removal was performed using the EEGLAB toolbox (Version 14.1.1b, Swartz Center for Computational Neuroscience) in Matlab (Version R2016b, The MathWorks, Inc., Natick, MA, United States) (Delorme and Makeig, 2004). Data were filtered using the EEGLAB filter function *pop_eegfiltnew*; high pass, 1 Hz; low pass, 30 Hz. The filtering option in EEGLAB uses linear finite impulse response (FIR) filtering with a second-order Hamming window. It was applied to the EEG signals to eliminate the noise from the subjects’ movement, breathing, and muscle electrical activity. After artifact removal, the signals were re-referenced with a common average of potentials at 61 electrodes, except for the ground and reference electrodes, by applying the *pop reref* algorithm. After the application of the common average re-reference (CAR) method, independent component analysis (ICA) was performed by applying the *pop runica* algorithm. After the ICA analysis, to remove the eye-blink noise, we removed components with a large weight (typically 1 and 2 components) in Fp1 and Fp2 while having an eye-blink pattern. A time-frequency representation was generated by a Morlet wavelet transform with a wavelet length of three cycles, a window size of 3000 ms, and a shift time of 100 ms (Akin, 2002). Four frequency bands were integrated: delta (1–4 Hz), theta (4–8 Hz), alpha (8–13 Hz), and beta (13–30 Hz). We focused on the theta, alpha, and beta bands in this study.

### EEG Analysis

A percentage value was used to normalize the individuals’ baseline, because the absolute amplitude of EEG oscillations is as dependent on skull conductivity and geometry as it is on neuronal dynamics. The change in the EEG power during the task was calculated as a relative change in the average EEG power between the gaming task and baseline, as follows:

$$P\,e\,r\,c\,e\,n\,t\,a\,g\,e\,v\,a\,l\,u\,e=\left(P\,o\,w\,e\,r\,\left(T\,a\,s\,k\right)-P\,o\,w\,e\,r\,\left(B\,a\,s\,e\,l\,i\,n\,e_{P\,R\,E}\right)\right)*\frac{100}{P\,o\,w\,e\,r\,\left(B\,a\,s\,e\,l\,i\,n\,e_{P\,R\,E}\right)}$$

**FIGURE 2:**
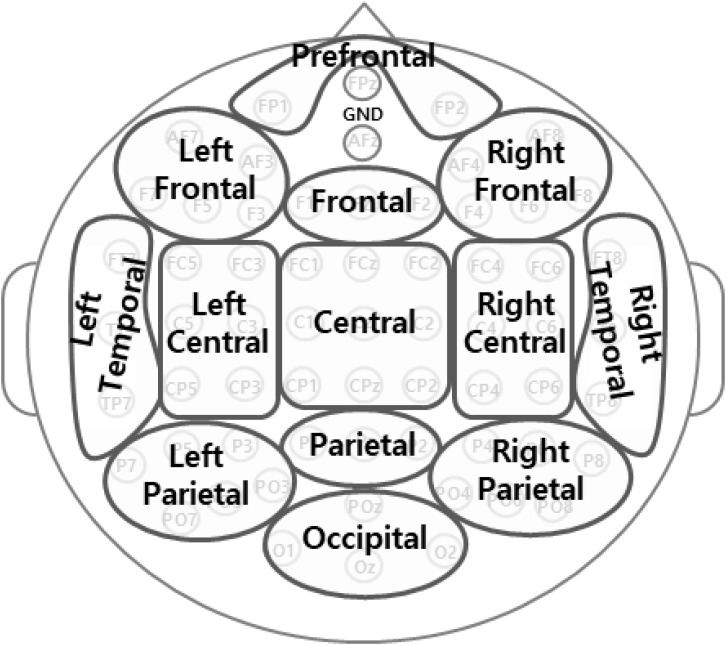
Representation of the 13 regions from 61 electrodes in the analyses.

As shown in Figure 2, we divided the activity at 61 sites into 13 regions by averaging within each region (according to the international 10-10 system): prefrontal (FP1, FP2), left frontal (F7, F3, AF7, AF3, F5), frontal (Fz, F1, F2), right frontal (F4, F8, AF4, AF8, F6), left central (FC5, C3, CP5, FC3, C5, CP3), central (FC1, FC2, Cz, CP1, CP2, FCz, C1, C2, CPz), right central (FC6, C4, CP6, FC4, C6, CP4), left temporal (T7, FT7, TP7), right temporal (T8, FT8, TP8), left parietal (P7, P3, P5, PO5, PO3, PO7), parietal (Pz, P1, P2), right parietal (P4, P8, P6, PO4, PO6, PO8), and occipital (POz, O1, Oz, O2).

### Statistical Analysis

Independent-sample *t*-tests were performed to compare the demographic, clinical, and behavioral variables between the two groups. The differences in the EEG data were analyzed by means of a mixed-model analysis of variance (mixed ANOVA). To evaluate the between-group differences in the EEG percentage values in the theta, alpha, and beta bands in 13 ROIs, we used the gaming period (during gaming sessions 1-3 and post) as the within-subjects factor, and the group (IGD or HC group) as the between-subjects factor. *Post hoc* comparisons were applied to determine whether the differences between the groups (independent *t*-tests) and between certain periods (paired *t*-tests) were statistically significant. To avoid multiple comparison problems including three bands and 13 ROIs, a false discovery rate (FDR) correction algorithm was applied to the *p*-values (Groppe et al., 2011). We used an alpha value of 0.05 (2-tailed significance) and an FDR value of 0.2 for the statistics that corresponded to a control of the maximum proportion of false positives among the rejected null hypotheses of <20% (Genovese et al., 2002). The *p*-values as well as corrected *p*-values were included as part of the results. In addition, to determining the relationship between the EEG percentage value in each band and the severity of IGD, we conducted Pearson’s correlation analysis between the left frontal percentage value and the YIAT score for each band. All statistical analyses were performed using SPSS software (version 21; IBM, Inc., NY, United States).

## Results

### Demographics, Clinical Characteristics, and Behavioral Results of the Subjects

The demographics, clinical characteristics, and behavioral results of the subjects are presented in Table 1. The groups did not differ in age or IQ. The subjects in the IGD group scored significantly higher in the tests for severity of the online game-related problems (YIAT: *t* = 12.088, *p* < 0.001; K-scale: *t* = 5.500, *p* < 0.001).

**TABLE 1:** Demographics, clinical characteristics, and behavioral results of the subjects.

**Variables**	**Internet gaming disorder group (*N* = 24)**	**Healthy control group (*N* = 35)**	***t***	***p*-value**
Demographic data				
Age (years)	23.08 (2.71)	23.20 (2.47)	–0.172	0.864
FSIQ	110.79 (15.36)	113.45 (11.22)	–0.770	0.444
Clinical data				
YIAT	63.28 (9.87)	34.28 (8.63)	12.088	<0.001
K-scale	86.04 (17.48)	62.14 (15.07)	5.500	<0.001
BDI	8.95 (7.93)	7.45 (5.08)	0.886	0.379
BAI	5.04 (4.72)	5.17 (5.50)	0.118	0.907
BIS	54.76 (8.55)	48.80 (6.13)	3.352	<0.005
AUDIT	11.52 (7.51)	9.51 (5.40)	1.112	0.271
WURS	24.70 (16.13)	23.85 (13.71)	0.218	0.828
Gaming time				
GAME1	31.50 (7.18)	29.65 (8.39)	0.877	0.384
GAME2	32.91 (10.74)	29.94 (7.22)	1.273	0.208
GAME3	29.29 (7.36)	30.42 (8.88)	–0.517	0.607
Time spent gaming	26.63 (24.39)	18.84 (15.64)	1.382	0.134
per week, hours				

*Data are presented as the mean (SD). FSIQ, full scale intelligence quotient; BDI, beck depression inventory; BAI, beck anxiety inventory; BIS, Barratt Impulsiveness Scale; AUDIT, alcohol use disorder identification test; WURS, Wender Utah Rating Scale for ADHD. Independent-sample *t*-tests were used.*

The two groups did not differ significantly in self-reported depression, anxiety, alcohol-related problems, and ADHD symptoms. However, the subjects in the IGD group scored significantly higher than those in the HC group in the tests for impulsivity (BIS: *t* = 3.352, *p* < 0.005). The gaming time during the experiment was approximately 30 min, and there was no difference between the two groups.

### EEG Activity

**FIGURE 3:**
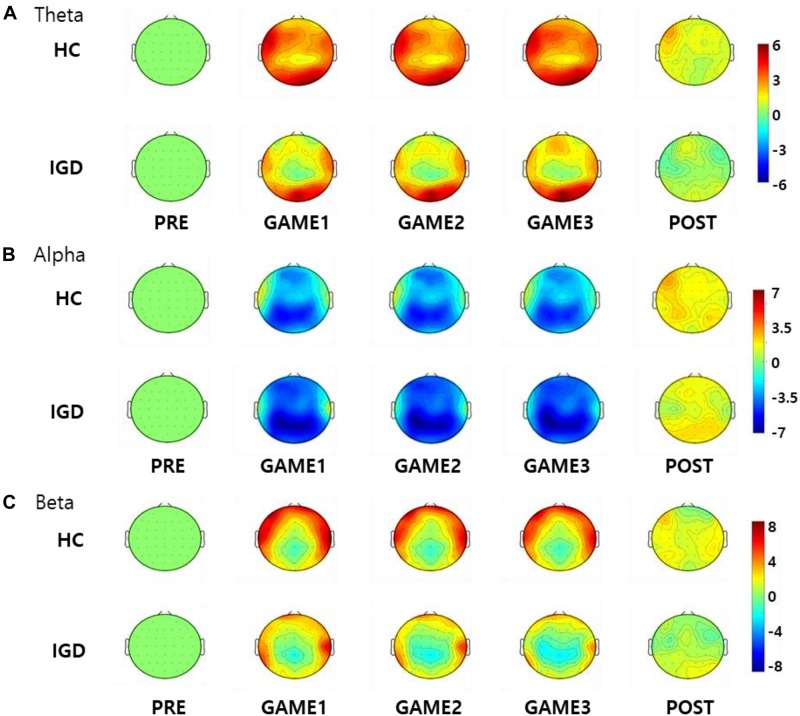
Topographical maps of the percentage values in the **(A)** theta, **(B)** alpha, and **(C)** beta bands in the healthy control (HC) subjects and those with Internet gaming disorder (IGD) during different periods (PRE, GAME1, GAME2, GAME3, and POST). Red represents a higher value, while blue represents a lower value. The IGD group demonstrated reduced percentage values in the theta, alpha, and beta bands compared to those in the HC group. Scales display% for percentage power.

**FIGURE 4:**
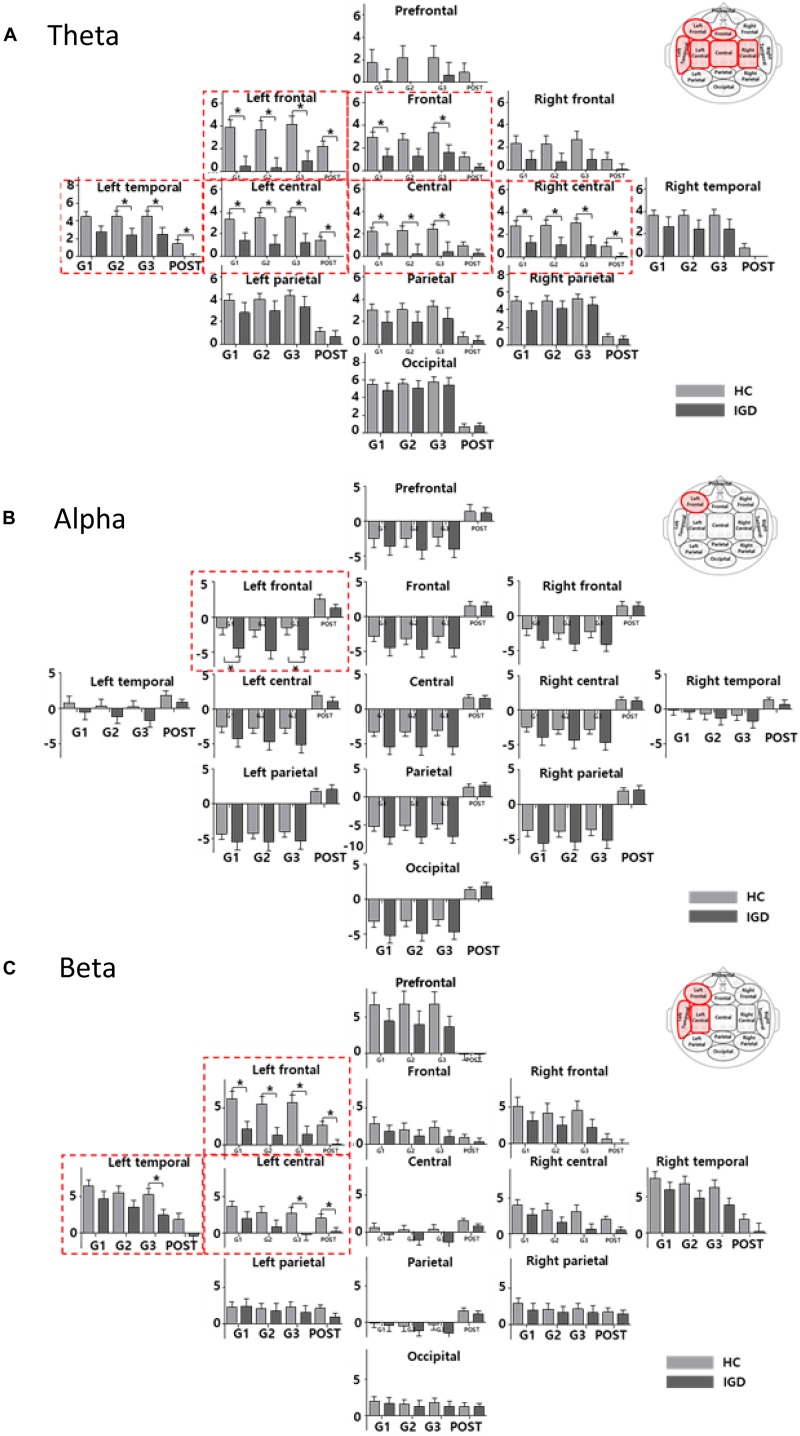
The bar graph represents the results of mixed ANOVA for the 13 regions in the **(A)** theta, **(B)** alpha, and **(C)** beta bands. The X-axis represents the period (GAME1, GAME2, GAME3, and POST) and Y-axis represents percentage values for the 13 regions (light gray: HC, dark gray: IGD). The bar graph is located upward at percentage values greater than 0 and downward at percentage values less than zero. The red box indicates a region with a significant difference for the group effect (mixed ANOVA; *p* < 0.05, FDR correction; corrected *p* < 0.2). The *post hoc* results from the independent *t*-tests between the groups are shown as asterisks (^∗^). Error bars indicate standard error. ^∗^*p* < 0.05.

Figure 3 displays the scalp topographies of the theta, alpha, and beta bands for the IGD and HC groups. For all bands analyzed by the mixed-model ANOVA, the main effect of the group was found to differ significantly between the groups (Figure 4). The data satisfied both normality and homogeneity of variance. In the theta band, left frontal: *F*(1,57) = 12.713, *p* = 0.001, corrected *p* = 0.039, effect size = 0.182; frontal: *F*(1,57) = 4.701, *p* = 0.034, corrected *p* = 0.165, effect size = 0.076; left central: *F*(1,57) = 8.707, *p* = 0.005, corrected *p* = 0.065, effect size = 0.133; central: *F*(1,57) = 6.610, *p* = 0.013, corrected *p* = 0.126, effect size = 0.104; right central: *F*(1,57) = 6.619, *p* = 0.013, corrected *p* = 0.101, effect size = 0.104; left temporal: *F*(1,57) = 5.318, *p* = 0.025, corrected *p* = 0.162, effect size = 0.085 (effect size as partial eta-squared). In the alpha band, left frontal: *F*(1,57) = 4.614, *p* = 0.036, corrected *p* = 0.156, effect size = 0.075. In the beta band, left frontal: *F*(1,57) = 10.702, *p* = 0.002, corrected *p* = 0.039, effect size = 0.158; left temporal: *F*(1,57) = 4.598, *p* = 0.036, corrected *p* = 0.1404, effect size = 0.078; left central: *F*(1,57) = 4.834, *p* = 0.032, corrected *p* = 0.178, effect size = 0.075 (mixed ANOVA; *p* value <0.05, FDR correction; corrected *p* value <0.2). The percentage values for all measured bands were lower in the IGD group than those in the HC group. For all bands, the left frontal region differed significantly between the groups.

We also found significant main effects for the period for all bands. The theta band differed significantly in 12 regions (every region except the prefrontal region). The alpha band differed significantly in all 13 regions and the beta band differed significantly in 10 regions (all but the left parietal, right parietal, and occipital regions). The interactions between the groups and the period were not statistically significant in any region.

### Correlation Analyses

**FIGURE 5:**
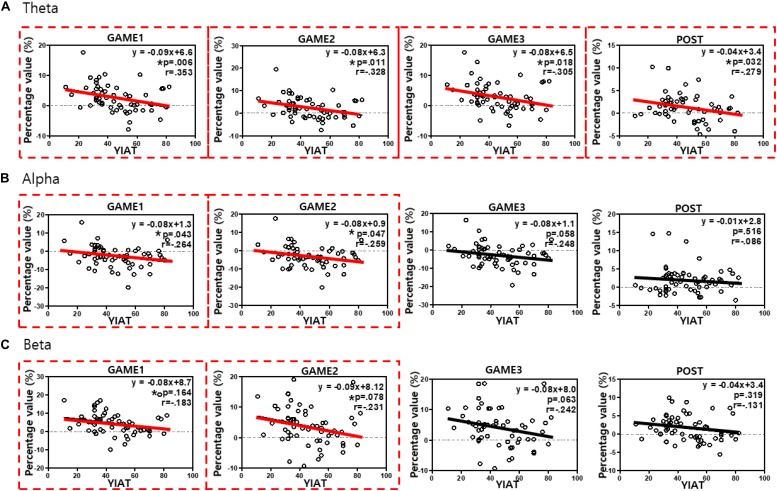
Correlation analyses between the YIAT score and the percentage value in the left frontal region in all subjects in the **(A)** theta, **(B)** alpha, and **(C)** beta bands. The red boxes indicate statistical significance (*p* < 0.05), indicating periods with a weak correlation between the YIAT score and percentage value in the left frontal region. Significant negative correlations were observed in all periods for the theta band, during GAME1 and GAME2 periods for the alpha band, and during GAME1 and GAME2 periods for the beta band. ^∗^*p* < 0.05.

To investigate whether the activity in the left frontal region could be a biomarker for IGD, we analyzed the relationship between the YIAT score and the power of each band in the left frontal region. We found significant negative correlations between the YIAT score and percentage value in the left frontal region in the theta, alpha, and beta bands in several periods. In the theta band, GAME1: *r* = −0.353, *p* = 0.006; GAME2: *r* = −0.328, *p* = 0.011; GAME3: *r* = −0.305, *p* = 0.018; POST: *r* = −0.279, *p* = 0.032 (Figure 5). In the alpha band, GAME1: *r* = −0.264, *p* = 0.043; GAME2: *r* = −0.259, *p* = 0.047. In the beta band, GAME1: *r* = −0.257, *p* = 0.049; GAME2: *r* = −0.263, *p* = 0.044.

## Discussion and Conclusion

We investigated the differences in the EEG response patterns in the theta, alpha, and beta bands between the HC and IGD groups during the gaming periods. We recruited individuals with IGD who exhibited only impulsivity, without other accompanying mental illnesses (depression, anxiety, alcohol use disorder [AUD], or ADHD). This is the first study to acquire real-time scalp EEG signals and analyzed longitudinal data during playing the online game LOL, the subjects’ primary game. The subjects with IGD had significantly lower theta, alpha, and beta band powers in the left frontal region than those of the HCs during gaming. The association between impaired cognitive control and IGD during gaming is represented by the lower frontal theta activity. This is associated with less cognitive control, whereas the lower frontal alpha activity suggests a lower load on the working memory. It is likely that validation and application of meaningful clinical indicators for IGD diagnosis and treatment will be possible through the addiction-related features of individuals with IGD that appear during gaming. In addition, the left frontal theta activity, which correlates with the severity of addiction, is likely to be used to discriminate individuals with IGD during gaming in real-time.

The frontal theta activity has been shown to be related to cognitive control (Brand et al., 2014; Cavanagh and Frank, 2014). Cognitive control is an executive function in the control system that allows us to regulate our behavior so that it is planned, goal-oriented, flexible, and effective (Shallice and Burgess, 1996; Jurado and Rosselli, 2007; Anderson et al., 2008). IGD is associated with impaired cognitive control, characterized as a behavioral addiction or impulse-control disorder (Dong et al., 2011, 2012, 2013). Additionally, increased alpha activity is related to the load on the working memory (Gundel and Wilson, 1992; Gevins et al., 1997; Klimesch, 2012). Katahira et al. (2018) suggested that the amplitudes of frontal theta and alpha activity were significantly smaller under conditions of boredom (accuracy 99.7%) than under flow conditions (accuracy 54.4%). Our results indicate that lower frontal theta activity can be interpreted as decreased cognitive control, while lower frontal alpha activity can be interpreted as a lower working memory load condition. Furthermore, it is assumed that the individuals with IGD are gaming under boredom conditions, characterized by lower frontal activity of both theta and alpha waves. The reduced frontal beta power in individuals with IGD is associated with impaired inhibitory control and reward feedback (Choi et al., 2013; Lee et al., 2014; HajiHosseini and Holroyd, 2015).

The left region of the brain is related to analytical and strategic thinking, and the LOL game requires strategic thinking. In this study, the HC group showed higher left frontal theta power than those in the IGD group during LOL gaming. In other words, based on the left frontal theta activity, it seems that the HC subjects performed the LOL game with more strategic thinking than individuals with IGD.

Imaging studies on addictive behavior have identified the key role of the prefrontal cortex (PFC), both through its regulation of the limbic reward regions and its involvement in higher-order executive functions (Goldstein and Volkow, 2011). PFC dysfunction is associated with activation of the limbic system, which results in addictive behavior (Crews and Boettiger, 2009). In other words, impaired executive functions contribute to an imbalance between the frontal cortical functions and a hyperactive limbic system that drives impulsive behavior (Bechara, 2005). Lee et al. reported that weaker dorsolateral prefrontal activation is correlated with higher cognitive impulsivity in IGD. The accuracy of the Stroop task was lower in the addiction group than in the normal group (Lee et al., 2015). Koepp et al. (1998) reported that the frontal striatal and limbic brain regions involved in the dopamine mesolimbic pathway are associated with the urge to play games and the reward mechanism.

In terms of reinforcement learning in a gaming environment, the player recognizes the current state and selects actions that maximize reward among the selectable actions (Montague, 1999). A feature of reinforcement learning is that when a player selects an action, the environment changes by his/her reactions to it. In the LOL game, it is difficult for a player to know what another player is thinking. There are those who strive to think strategically and obtain information based on the characteristics of the LOL game; however, there are also those who seek only immediate rewards. Players who only pursue immediate rewards are reward-seeking, and this can easily lead to addiction. In the brain, goal-directed control predicts a future situation based on the given information, establishes a strategy to achieve the goal, and acts as planned. In contrast, stimulus-driven control is not based on the given information and the participant responds to the situation without strategy or planning (Corbetta and Shulman, 2002). Repetitive stimulus-driven control results in individuals losing control over their gaming behavior with a loss of control over gaming.

This study has several limitations. First, as the scalp EEG was recorded during real-time online gaming, it is possible that there was noise in the EEG signals due to physical movements (i.e., wrist movement, neck motion). To reduce this effect, we instructed subjects not to engage in activities unrelated to gaming. Additionally, various filters were used to remove the noise from the data in the analytic processes. Future studies should consider additional biological signals that can reflect the subject’s physical condition (i.e., EMG, PPG), which can be effective tools for establishing objective indicators during gaming. In addition, only male subjects were included in this study, which is the reason that generalization of the results of the current study may be limited. Finally, the changes in the scalp EEG signals in response to specific game events were not evaluated in this study. Future studies, including an analysis of the behavioral characteristics of individuals with IGD during specific game events and their accompanying scalp EEG signal responses, may expand on our present study.

There are several strengths associated with this study. First, to the best of our knowledge, this is the first study to compare EEG activity recorded in individuals with IGD and HC subjects during gaming. Most previous studies reported EEG activity measured in the resting state, but we concluded that it is important to observe the reaction of participants to gaming cues in order to understand the addiction (Choi et al., 2013; Lee et al., 2014; Park et al., 2017). In addition, we made considerable efforts to recruit subjects who only exhibited impulsivity, in order to control for other factors. Almost all previous studies conducted in patients with IGD included subjects who had comorbid disorders (depression, anxiety, AUD, and ADHD).

In summary, based on the findings reported here we conclude that an association exists between impaired cognitive control and IGD during gaming. Our data demonstrated that individuals with IGD had significantly lower theta, alpha, and beta power in the left frontal region than that of the HCs while gaming. The collective data on theta, alpha, and beta activity suggest that the low theta and alpha activity indicated a boredom state, while the low beta activity reflected impulsivity and impaired inhibitory control during gaming. In addition, the degree of left frontal theta power suppression was found to be correlated with the severity of IGD during gaming. These findings suggest that reduced percentage values for the responses in the theta, alpha, and beta bands in the left frontal region during gaming have the potential to be neurobiological markers for IGD.

## Ethics Statement

All subjects provided written informed consent before beginning with the experiment. Moreover, the experimental protocols were approved by the Institutional Review Board of Hanyang University (IRB number: HYI-16-044).

## Author Contributions

JK, JP, YP, DJ, KN, Y-CJ, and IK conceived and designed the experiments. JK and YP conducted the experiments. JP and DJ analyzed the data. KN, YJ, and IK wrote the manuscript. All authors revised and approved the manuscript.

## Conflict of Interest

The authors declare that the research was conducted in the absence of any commercial or financial relationships that could be construed as a potential conflict of interest.
